# Case of Abscess of the Lung Cured by Incision and Drainage

**Published:** 1885-03-20

**Authors:** T. Pridgie Teale

**Affiliations:** Consulting Surgeon to the General Infirmary at Leeds


### CASE OF ABSCESS OF THE LUNG CURED BY INCISION AND DRAINAGE.

By T. PRIDGIE TEALE, M. B., F. R. C. S., ENG.,
Consulting Surgeon to the General Infirmary at Leeds.

The following case is interesting as a contribution to the discussion which has been raised on a very important question :

On March 1st, 1881, I was requested to visit Mr. B———, aged fifty-four, in consultation with Mr. Ireland, of Tadcaster, and to tap a pleuritic effusion on the right side of the chest. The patient had been ill for three months, and had been seen occasionally by the late Dr. Shann of York, Dr. Clifford Allbutt of Leeds, and Dr. Myrtle of Harrogate. The earlier history of the case had been most obscure, commencing in December, 1880, with a cold, shivering, loss of flesh, vomiting and retching, and pain in the hepatic region, but there was no cough, nor any symptom of lung disease. Dr. Allbutt, in the middle of February, found the hepatic dullness increasing upward, as if there were fluid in the lower part of the thorax, and a week later (February 28) Dr. Myrtle saw the patient, and, finding dullness occupying the lower half of the right lung, came to the conclusion that fluid was present, and advised early tapping.

On March 1, 1881, after examining the chest, I came to the conclusion as the other medical men—namely, that there was fluid in the pleural cavity. The right side of the thorax having been punctured low down with the small trocar of Bartleet's aspirator, a few drops of clear straw-colored fluid escaped, and even after the addition of the suction of the aspirator only two drachms of fluid were obtained. Being still confident that fluid was present, I made a fresh puncture higher up, when very offensive, thin, greenish pus appeared, but only in drops. By applying the aspirator, more pus was withdrawn, and, after careful and continuous aspiration, about a pint of pus was slowly obtained. My view at the time was that there were two separate collections of fluid in the pleural cavity, shut off by adhesion from one another. In consequence of the unexpected character of the case, two days later a consultation was held with Dr. Allbutt, Dr. Myrtle, and Mr. Ireland, and it was decided that as soon as the temporary relief given by the tapping had passed by, the thorax should be opened and drained.

On the 16th the patient had become more hectic, had expectorated for two days most offensive pus, and was altogether extremely ill. The following notes of Mr. B———'s condition were taken at the time by Mr. R. N. Hurtley : The chest as a whole was rather rigid, moving little during respiration, with perhaps slight inequality of movement of the two sides. The respiration was tranquil (30) ; the pulse 150. The left side was normal. On the right side there was continuous dullness below and over the hepatic area, extending upwrad in front to about the level of the fifth rib, a little below the nipple, and laterally round the axilla, and diminishing toward the spine. In the recumbent posture there was a spot in the front of the chest, having a peculiar "cracked pot" resonance, in the middle of a dull area. The area of the dullness was somewhat altered by change of position. Breathing but faintly audible over the dull area. No rales.

### OPERATION.

An exploring trocar was introduced at a point a little below and in front of the angle of the scapula (ninth intercostal space), and a syringeful of fetid pus was withdrawn. The patient having been very cautiously put under ether by Mr. Hartley, I made, with the assistance of Mr. Ireland, an incision at the site of the puncture, and opened the pleural cavity. No pus appeared; only a small quantity of serum. The finger introduced into the pleural sac discovered the lung, but no large space. The adjacent pleural surfaces were rough, and numerous adhesions were broken down easily by the finger passing in all directions. The lung felt dense and boggy, not crepitant and elastic. On reintroducing the trocar and puncturing the lung, pus appeared. The puncture was enlarged so as to admit the finger, and two pints of the most fetid pus escaped, rendering the room almost unbearable. A large drainage tube, about six inches long, having been inserted, the cavity was syringed out with a weak solution of carbolic acid, and the chest was encased in carbolic tow, etc.

The after process of the case was extremely tedious and critical. As far as the local condition in the chest was concerned, the treatment was comparatively simple. The cavity in the lung was washed out (at first three times a day) with a weak solution of carbolic acid or Condy's fluid, by means of a small catheter introduced along the drainage tube. For three weeks the offensive character of the discharge continued, but at the end of this time it was comparatively sweet, though considerable in quantity. On one occasion the patient expectorated a little pus, but this ceased spontaneously, and, with the exception of an occasional "suffocating feeling" when the cavity was being syringed out, symptoms directly referable to the lung mischief were not prominent. The patient's general condition, however, was for months one of the greatest anxiety, the symptoms being entirely those of septicæmic poisoning. Persistent vomiting, hectic, profuse sweating, delirium, and obstinate hiccough (lasting at one time almost incessantly for a whole week) were the most prominent features of the illness. At the beginning of July an attempt was made to move the patient to Scarborough, but was abandoned in consequence of a prolonged faint as soon as he was placed in the carriage. Later on, after a critical and dangerous attack of diarrhœa, the tongue cleaned, and he began to improve, and to be able, almost for the first time for months, to assimilate an appreciable quantity of food at a time. The discharge from the sinus in the lung had all this time been gradually lessening, and the drainage tube, which had been shortened at the rate of about an inch per month, was expelled spontaneously about August 24th, and could not be replaced, the sinus finally healing a few days afterwards. Before Christmas—i. e. within a year of the commencement of his illness, and within nine months of the incision into the lung—the patient had resumed his active work as a solicitor.

REMARKS.

1. The surgical treatment of affections of the lung being, as yet, in a tentative stage, I have thought it well to report this case somewhat fully, as it is instructive in many points, is complete in its results, and, considering the age of the patient, is rare, if not unique.

2. Although in commencing the operation I thought the case to be one of empyema, my course would have been the same had I believed that the pus came from a cavity in the lung. It is clear, also, that the fact of the offensiveness of the pus ought to have suggested to me the probability that the collection was in the lung, and not in the pleural cavity.

3. The early history of the affection was most obscure. It seems not impossible that it commenced as a diaphragmatic pleurisy with a circumscribed empyema, which made its way upward into the pulmonary tissue.

4. Another instructive point is this : that the first puncture with a small trocar only withdrew, even when aided by the aspirator, two drachms of serum ; and that the pus was discovered by a second puncture. The pus, also, could not be withdrawn without the aid of the aspirator.

5. The pus case illustrates the importance of having an aspirator at hand when tapping a chest, to be used *only* in case the fluid will not flow spontaneously through the cannula and attached India rubber tube. Barttleet's pocket aspirator is most convenient and useful, and does not apply suction too powerfully.

6. The question, whether the opposed layers of pleura are adherent hardly affectd the decision of the surgeon, chiefly because in the cases calling for interference it is impossible to ascertain the point, except by operation ; secondly, because in suitable cases, in which, as a rule, there will be a large area of dullness, it seems probable that pneumothorax is not one of the factors that will arise to impair the prospects of success. Two cases of traumatic pneumothorax—not from broken rib—with healthy lung, under my care, have made good and rapid recovery.

7. The case is an additional encouragement to relieve surgically abscess and gangrene of lung. Such attempts will probably be most successful when the abscess can be reached and drained posteriorly through the ninth or tenth intercostal space.—*Lancet*, *July 5, 1884.*

---

**Phosphate of Zinc in Dysmenorrhœa.**—Decoux ("Union Med.") writes in praise of this drug, which he has used in many cases of dysmenorrhœa and amenorrhœa, with satisfactory results. He gives a little less than a third of a grain daily—two pills of about a twelfth of a grain each before breakfast, and two more at night. He admits that this drug possesses no distinctive properties other than those of pure phosphorus, but thinks that it is safer and more manageable.—*N. Y. Med. Jour.*

